# Androgen increases klotho expression via the androgen receptor-mediated pathway to induce GCs apoptosis

**DOI:** 10.1186/s13048-022-01087-w

**Published:** 2023-01-14

**Authors:** Xin Zeng, Qiaoqing Zhong, Ming Li, Yating Liu, Shuanglian long, Yuanjie Xie, Zhongcheng Mo

**Affiliations:** 1 Guangxi Key Laboratory of Diabetic Systems Medicine, Guangxi Province Postgraduate Co-Training Base for Cooperative Innovation in Basic Medicine, Guilin Medical University, Guilin, 541199 Guangxi China; 2 Department of Basic Medicine, Chongqing College of Traditional Chinese Medicine, Chongqing, 402760 People’s Republic of China; 3 Department of Cardiovascular Medicine, Xiangya Hospital, Central South University, Changsha, 410008 China; 4 Department of Anesthesia, Critical Care & Pain Med, Beth Israel Deaconess Medical Center, Harvard Medical School, Boston, MA 02115 USA; 5 Clinical Anatomy & Reproductive Medicine Application Institute, Hengyang Medical School, University of South China, Hengyang, 421001 China

**Keywords:** Testosterone propionate, Androgen receptor, Klotho, Polycystic ovarian syndrome

## Abstract

**Background:**

Many epidemiological studies have shown that anovulatory polycystic ovary syndrome (PCOS) is accompanied by hyperandrogenism. However, the exact mechanism of hyperandrogen-induced anovulation remains to be elucidated. In this study, we aimed to investigate the potential mechanism of anovulation in PCOS. To investigate the role of klotho as a key factor in the androgen receptor (AR)-mediated development of PCOS, we investigated the effects of testosterone on ovarian klotho expression in vivo and in vitro.

**Results:**

Testosterone propionate (TP)-induced rats showed cycle irregularity, hyperandrogenism, polycystic ovarian changes, dyslipidemia. However, inhibition of AR expression could relieve PCOS traits. We also found that AR and klotho showed relatively high expression in PCOS rat ovarian tissue and in TP-induced granulosa cells (GCs), which was inhibited by the addition of flutamide. TP-induced GCs apoptosis was suppressed by AR antagonist, as well as silencing klotho expression in human GCs. Chromatin immunoprecipitation assay demonstrated that AR indirectly binds to the klotho promoter.

**Conclusions:**

Our results demonstrated TP mediates the expression of klotho via androgen receptor and klotho alterations could be a reason for ovarian dysfunction in PCOS.

## Introduction

PCOS is a heterogeneous disease that is mainly characterized by hyperandrogenism, ovulation changes, infertility and polycystic ovaries [1]. Abnormal folliculogenesis is considered the main cause of anovulation. In PCOS patients, B-mode ultrasound often shows polycystic changes in the ovary, and the transformation of sinus follicles to mature follicles is arrested [2]. Follicle development disorder is proposed to be closely related to apoptosis of GCs. Apoptosis of GCs was observed in both animal models of PCOS and PCOS patients [3, 4]. Therefore, GCs have been extensively studied in the pathogenesis of PCOS. It has been found that the pathological mechanism of GCs apoptosis promoting the development of PCOS may be related to sex hormones, steroid hormones, genetic factors, mitochondrial dysfunction, hypothalamic-pituitary-ovarian axis imbalance etc. [3–6]. Among these studies, the effect of androgens on GCs has been widely concerned, because the level of androgens determines whether the follicle successfully develops to maturity. As the most typical clinical manifestation of PCOS, hyperandrogenemia is worth exploring the potential pathogenesis between it and GCs apoptosis.

Excess androgen not only causes hairy and acne but also it increases over-recruitment of follicles, which leads to the absence of dominant follicles, ultimately resulting in anovulation [7]. In fact, androgens also play a significant role in follicle development. It has been reported that the characteristics of androgen regulation in ovarian follicle development depend on the follicular phase [2]. During folliculogenesis, androgens can inhibit antral follicle growth. By contrast, preantral follicles respond to androgens by promoting follicular growth [8]. Long-term hyperandrogenic state of PCOS patients is one of the reasons leading to anovulation, while follicular dysplasia may be caused by abnormal apoptosis of GCs. Previous results have demonstrated that androgen excess induced the apoptosis of GCs. Numerous factors such as microRNA, inflammation and obesity may be involved in androgen-induced apoptosis [4, 9–11]. However, the underlying mechanism is still not clear. Androgen should conjunct with androgen AR to function. The role and regulation of AR in regulating androgen signaling during follicle development still need to be explored. Therefore, we used the androgen induced PCOS model to study AR mediated signaling pathways, and testosterone propionate, as a biologic agent of testosterone that exerts its effects after injection into the body, is a well-established model of induced PCOS.

AR is a steroid receptor in the nuclear receptor superfamily. AR expression has been identified in ovarian follicles in different stages but exhibits differential expression among the different stages [7, 12]. However, overall, the levels of androgen receptors in GCs from women with PCOS are significantly increased [13]. Knockdown of AR can decrease testosterone-induced granulosa-lutein cell apoptosis by downregulating DR5 and C/EBP homologous protein (CHOP) expression [14]. Kirsty A. Walters and colleagues found that in granulosa cell-specific AR knockout mice, there were significantly prolonged estrous cycles and decreased the number of pups born [15]. AR absence leads to severe damage to the hypothalamic-pituitary–gonadal (HPG) axis function and impairs follicular development [5]. These results reflect AR-mediated actions associated with key processes in the female reproductive tract. In renal tissue, androgen-activated AR can specifically bind to the androgen response element within the klotho promoter [16].

Klotho, a recently discovered aging suppressor gene, is expressed only in the brain, kidney, reproductive organs, pituitary gland, and parathyroid gland [17]. Previous studies have focused on the effect of klotho in the kidney, brain and tumor tissue [16, 18]. However, the effect of klotho on reproductive system is nonignorable. Compared with normal porcine cell lines, porcine cell lines overexpressing human klotho (ℎKlotho) or tetracycline (Tet)-inducible ℎKlotho showed an obviously higher blastocyst formation frequency [19]. In rats, lack of β-Klotho displayed growth reduced such as delayed puberty and subfertility via hypothalamic defect [20]. Notably, klotho seems to be associated with the underlying mechanism of PCOS and is positively correlated with apoptosis of ovarian granulosa cells in PCOS [21]. The development of GCs determines the fate of follicles. The mechanism of GCs apoptosis induced by hyperandrogenism in PCOS needs further clarification to determine whether klotho is involved.

In the present study, we aim to investigate whether TP changes the expression of klotho via AR-mediated pathway and is able to change reproductive functions in female rats and human ovarian granulosa cells. Thus, we evaluated several peripheral biomarkers, as well as the expression of AR and klotho, between TP group and control group. To further confirm the roles of AR and klotho, we observed the changes in KGN cell apoptosis induced by using androgen receptor antagonists and klotho silencing, and the ChIP experiment verified the binding between AR and klotho. This study provides a more scientific basis for establishing AR and Klotho as new targets affecting GCs apoptosis.

## Result

### Chronic androgen treatment induces follicular development arrest in rats

**Fig. 1:**
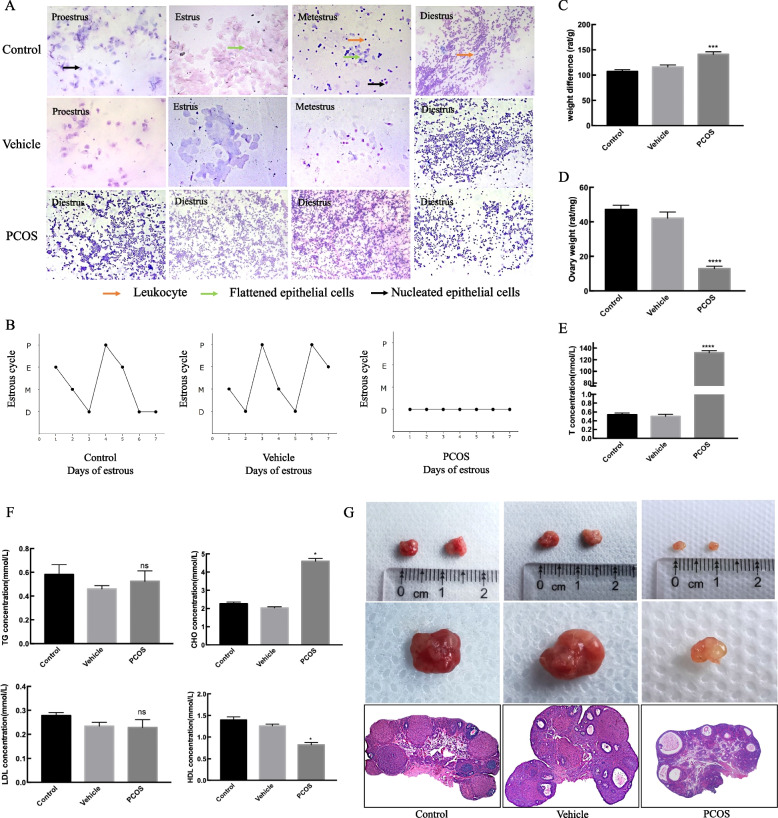
Chronic androgen treatment induces PCOS model. vaginal smear (**A**, **B**), body weight (**C**), ovary/body weight ratio (**D**), androgen level (**E**), blood lipid level (**F**), macroscopic view of ovary and HE staining (**G**) were used to detect the effect of TP in rats

We started using vaginal smear to monitor the estrous cycle after TP injection for 21 days. The results showed that TP-induced rats did not observe the complete estrus cycle and most of them stayed at diestrus (Fig. 1). To confirm the modeling situation, we tested the rat body weight, ovary/body weight ratio, androgen level, blood lipid level and HE staining (Fig. 1). We first observed the effect of chronic androgen treatment on body weight, ovarian morphology and serum biochemistry parameters in rats. We have obviously found that TP rats had higher body weight gain difference (*P* < 0.01 vs. control), lower ratio of ovary/body weight (*P* < 0.01 vs. control) and higher androgen level than control group (*P* < 0.001 vs. control). HE staining showed that ovaries of TP rats generally abundant corpus lutea, indicating a polycystic state. In contrast, a complete follicular cycle was observed in the control group. Taken together, these findings indicated that chronic androgen exposure is a negative regulator of ovarian growth and ovulation in rats, and can induce the PCOS model.

### Flutamide relieves abnormal ovulation in TP-induced rats

**Fig. 2:**
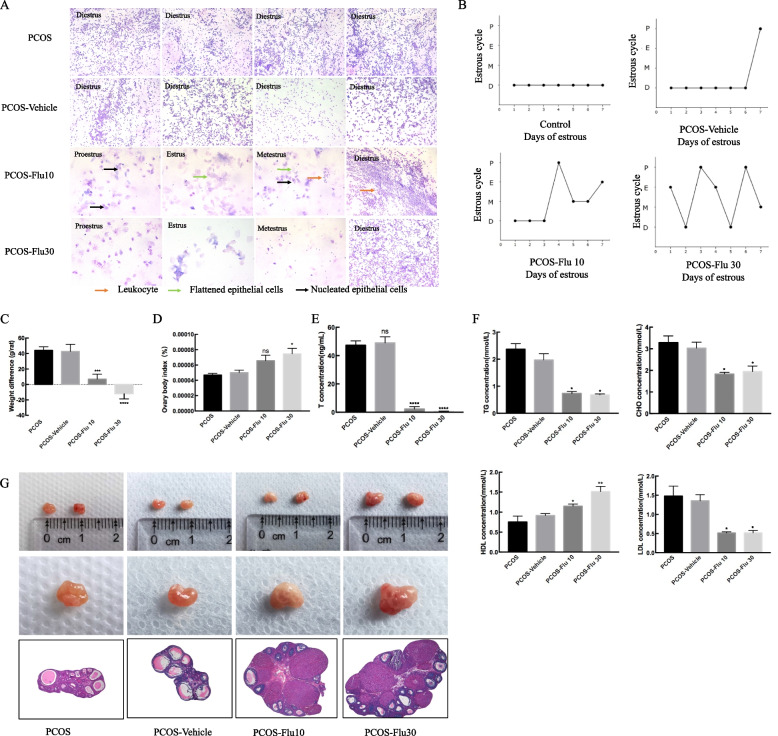
Flutamide improve the process of TP-induced PCOS model. vaginal smear (**A**, **B**), body weight (**C**), ovary/body weight ratio (**D**), androgen level (**E**), blood lipid level (**F**), macroscopic view of ovary and HE staining (**G**) were used to detect the effect of flutamide in TP-stimulated PCOS rats

To understand the effect of blocking AR expression on TP-treated rats. We treated PCOS rats with flutamide for 15 days, results showed that PCOS rats observe complete follicular cycle suggesting that ovaries restore ovulation (Fig. 2). Although the ovary/body weight ratio still did not recover to the normal level, the weight was obviously decreased (*** *P* < 0.005 vs. control, **** *P* < 0.001 vs. control). In the blood, the T level was significantly reduced (**** *P* < 0.001 vs. control) and dyslipidemia was improved. HE staining revealed the corpus luteum and increased number of GCs (Fig. 2). Our findings indicate that blocking AR expression can alleviate TP-induced PCOS symptoms.

### AR and klotho contents are increased in TP-induced PCOS rats

**Fig. 3:**
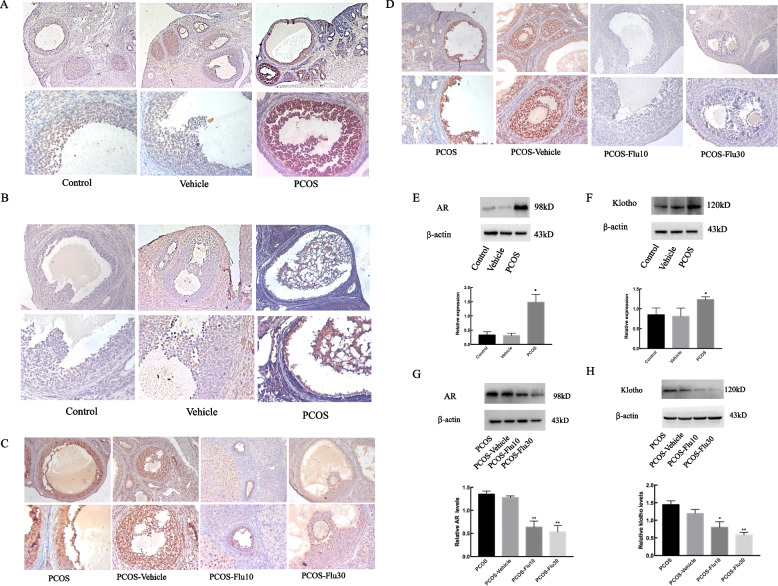
Androgen stimulates AR and klotho expression in ovary but flutamide can converse this situation Immunohistochemistry (**A**, **B**), Western blot (**E**, **F**) were used to detect the expression of AR in ovary. The results showed that the expression of AR and klotho were increased. However, using flutamide treatment for 15 days, both Immunohistochemistry (**C**, **D**) and Western blot (**G**, **H**) were showed the decreased expression of AR and klotho. **G** ** *P* < 0.01 vs. Control; **H** * *P* < 0.05 vs. Control, ** *P* < 0.01 vs. Control

To investigate how AR and klotho are affected after TP-treated rats for 35 days, we tested the expression of AR and klotho with Immunohistochemistry (IHC) and Western blot in TP-induced PCOS rats. In IHC, the expression of AR and klotho in the ovaries of TP-stimulated rats were up-regulated compared with the control group (Fig. 3). Western blot showed significantly increased AR protein expression in ovaries from TP-stimulated rats compared with those from the control group (Fig. 3E, *P* < 0.05). Klotho also showed high expression in PCOS (Fig. 3F, *P* < 0.05). However, flutamide could converse this situation (Fig. 3). Our findings suggested that androgen plays an important role in AR expression involved in the regulation of klotho in the ovary.

### TP inhibits granulosa cell viability and induces apoptosis

**Fig. 4:**
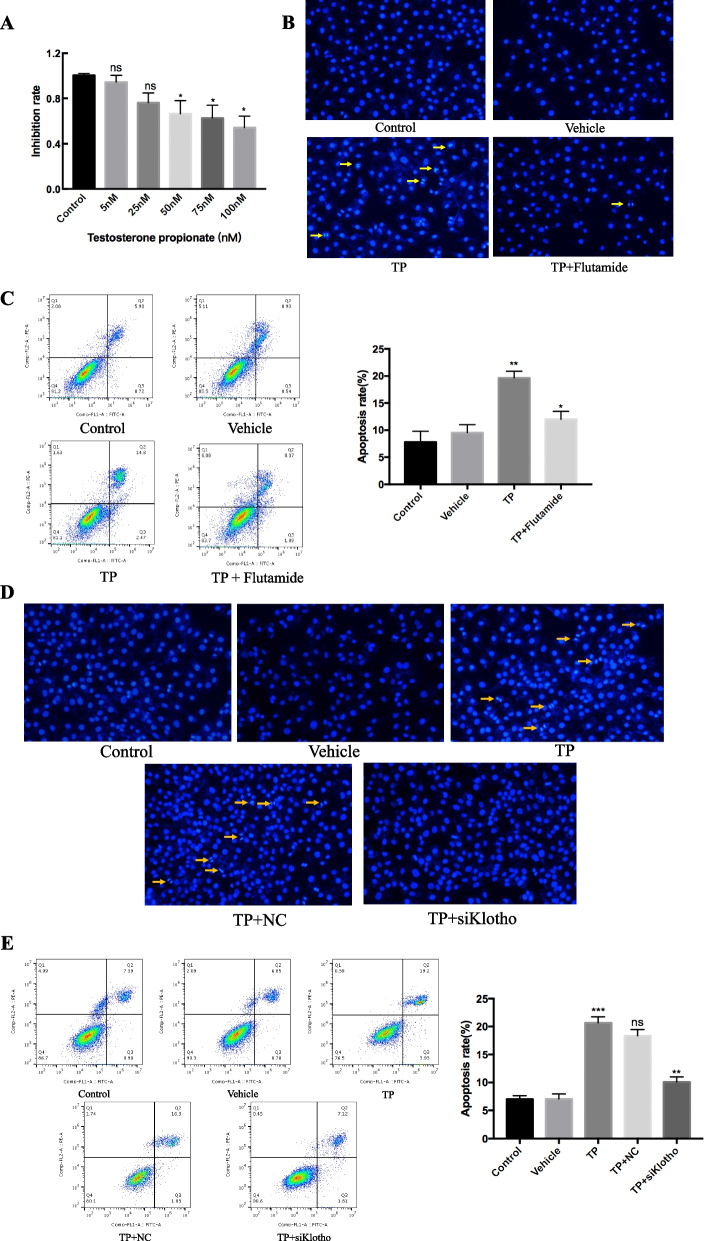
TP induces cellular apoptosis of granulosa cell. After intervening AR expression, CCK-8 (**A**, * *P* < 0.05 vs. Control), Hoechst (**B**, apoptotic bodies are indicated by yellow arrows, 20 ×) and Flow cytometry (**C**, ** *P* < 0.01 vs. Control, * *P* < 0.05 vs TP) were detected the viability and apoptosis respectively. To further verify the effect of klotho in TP-induced GCs, after silence klotho, also used Hoechst (**D**, apoptotic bodies are indicated by yellow arrows, 20 ×) and Flow cytometry (**E**, *** *P* < 0.001 vs. Control, ns *P* > 0.05 vs. TP, ** *P* < 0.01 vs. TP) to detected the GCs apoptosis

CCK-8 assay was used to assess cellular viability. The results showed that 50 nmol/L, 75 nmol/L and 100 nmol/L TP reduced the viability of GCs (Fig. 4, *P* < 0.05). Hoechst detected apoptosis in TP-stimulated GCs. Cells were seeded in 6-well plates in DMEM with FBS. TP (5 nmol/L, 25 nmol/L, 50 nmol/L, 75 nmol/L or 100 nmol/L) was added to incubate cells for 24 h. Subsequently, we combined with TP- and flutamide-treated GCs for 24 h. We found that TP increased GCs apoptosis, but the specific AR antagonist flutamide decreased TP-induced GCs apoptosis (Fig. 4B/C). To further investigate the role of klotho in TP-induced apoptosis of GCs, we added klotho silencing (siRNA) in TP-treated GCs, this response was significantly attenuated by klotho silencing (Fig. 4D/E).

### TP-induced GCs growth arrest is associated with increased AR and klotho

**Fig. 5:**
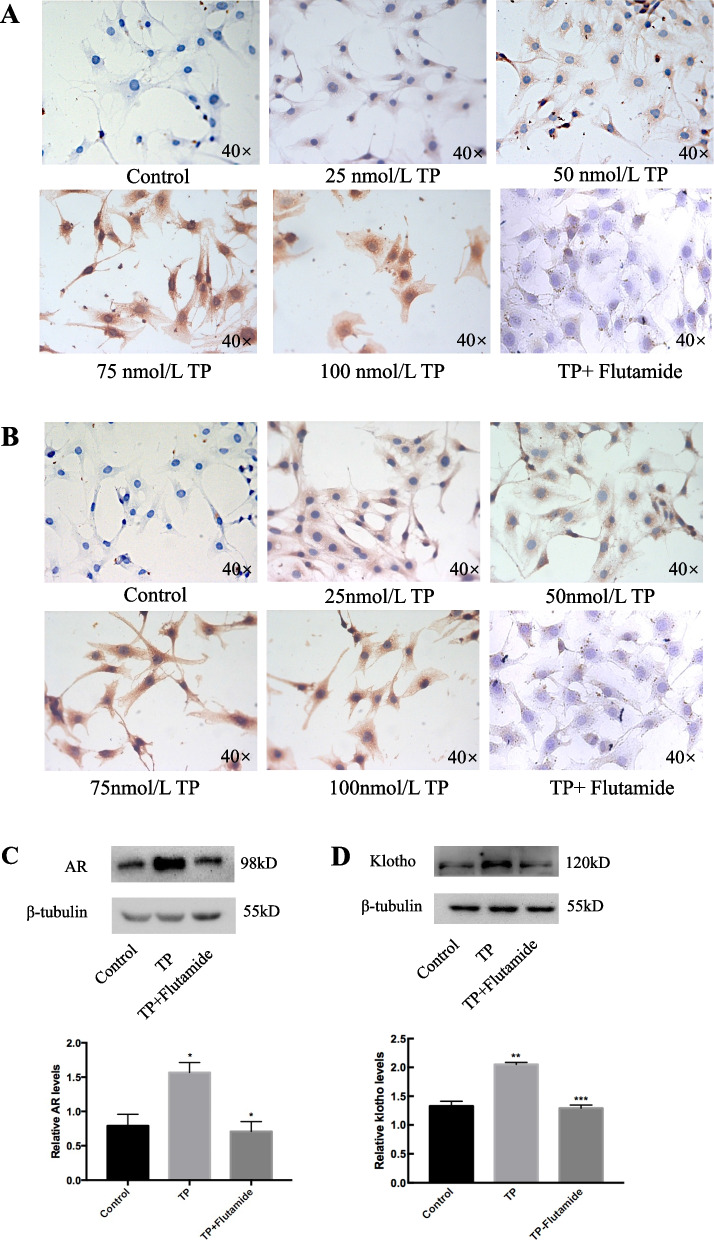
TP increases AR and klotho contents in vitro. GCs were cultured with TP (0—100 nM, 24 h) changes in AR and klotho expression were assessed by Immunocytochemistry (**A** and **B**, respectively). Western blot (**C** and **D**, respectively) was used to detect the expression of AR and klotho in TP (100 nM, 24 h)-induced GCs. The expression of AR and klotho were high-express in TP, this response was attenuated by flutamide

To explore the relationship among TP-induced GCs apoptosis, AR, and klotho. The expression of AR and klotho were detected by immunohistochemistry after TP treatment. The results showed that different concentrations of TP all promoted the expression of AR and klotho (Fig. 5). The expression of klotho was enhanced by overexpression of AR. Conversely, in the presence of flutamide, TP-induced klotho expression was attenuated (Fig. 5B). Western blot also showed that flutamide decreased the expression of klotho (#, *P* < 0.05 vs. TP). These results suggested that klotho affects TP-induced apoptosis of GCs mediated through AR.

### AR indirectly alters klotho expression to induce GCs apoptosis

**Fig. 6:**
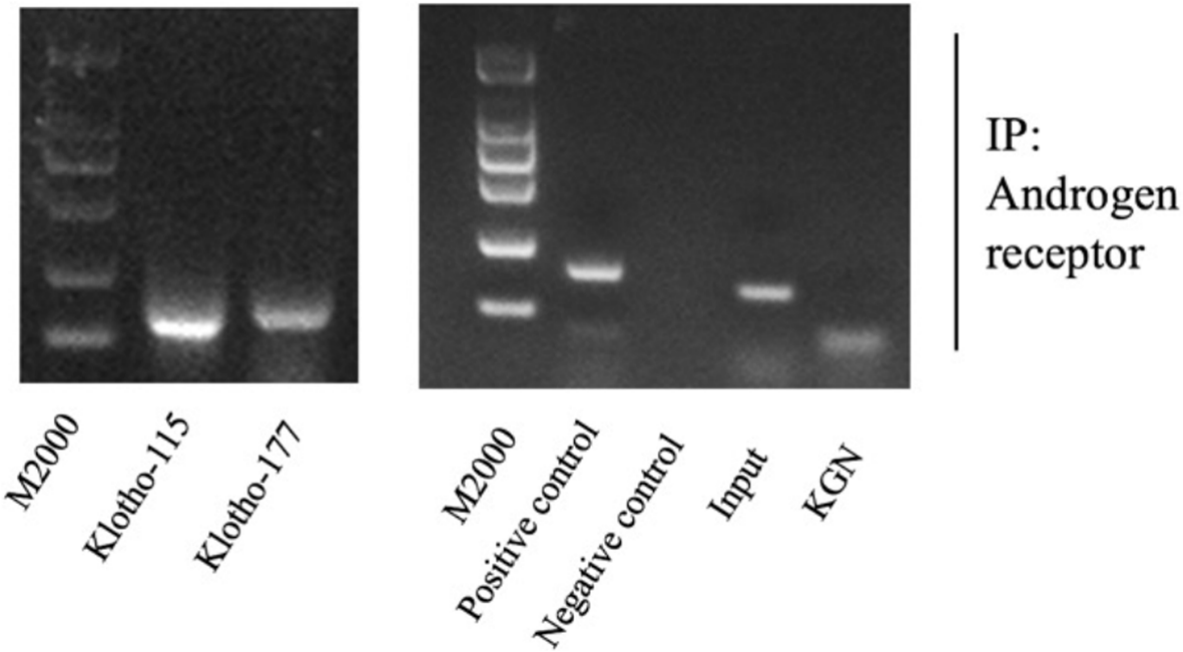
AR indirectly binds to klotho promoter

To clarify whether AR and klotho can be directly combined, we used the ChIP experiment for verification. According to the results of the primer pre-experiment, the primer klotho-115 was selected for subsequent formal experiments. The bands of the positive control group were clear and free of miscellaneous bands, and the negative control group had no bands, indicating that the ChIP experiment results were reliable and the operation was correct. The bands in the input group were clearly visible, indicating that the PCR amplification primers could be used to detect this indicator. The KGN experimental group had no bands at the corresponding positions, indicating that the promoter region of the klotho gene did not bind to AR in KGN cells, suggesting that TP indirectly regulates klotho expression through AR (Fig. 6).

## Discussion

Hyperandrogenism affects the apoptosis of GCs, which determines the fate of follicles. Androgen acts via the AR to regulate the development of follicular. Since AR can also be regulated by a variety of factors, the androgen/AR signaling that regulates follicular development in PCOS remains to be fully elucidated. In this study, we investigated that the mechanism of AR-mediated apoptosis of GCs. After successful construction of the PCOS model in this experiment, we used the AR-specific antagonist flutamide to interfere with AR expression. Compared with normal group, WB and IHC showed that upregulation AR and klotho expression in PCOS, conversely, the androgen-induced high expression of AR and klotho were attenuated after treatment with flutamide. In vitro experiments further confirmed that androgen induced GCs apoptosis is positively correlated with upregulation of AR and klotho expression, whereas in CHIP experiments it was found that AR does not directly act on klotho, suggesting that we can further explore the upstream and downstream targets of klotho.

Results have been inconsistent in a limited number of investigations klotho levels in women with PCOS. In our study, klotho expression was upregulated in the ovaries of PCOS, and some investigators have also found higher levels of klotho in women with PCOS than in heathy women [3, 22, 23]. Bednarska et al. reported higher serum b-klotho levels in 67 normal weight, young women with PCOS than in 18 fertile age women [22]. Gateve et al., in their study, also found similar serum levels of a-klotho comparing the PCOS group to controls [23]. In this study, we also found that dyslipidemia was improved after klotho was reduced. Based on the above experimental results, the increased expression of klotho in PCOS may not only affect follicle development but also be related to lipid metabolism, insulin resistance, obesity, etc. This provides some clues and ideas for the future research direction.

To reveal the exact role of the androgen/AR pathway in PCOS, it is necessary to replicate an animal model similar to human PCOS-like symptoms. According to the literature, several methods for constructing PCOS rat models have been explored, such as the use of androgen, letrozole, and HCG hormone modeling methods [24–26]. The most commonly used methods are the androgen and letrozole models. These two modeling methods have their advantages. They can both induce hyperandrogenism in rats, and polycystic changes in the ovary will occur. However, letrozole induces ovarian volume increase in rats, and androgens reduce ovarian volume. In terms of morphological and histological changes, rat models established with letrozole are more suitable for the study of ovarian morphology. But in order to study the androgen/AR signaling pathway and to eliminate the interference of letrozole on AR expression, we chose to use androgen for modeling.

Although we used TP-reduced rat model to study the relationship between GCs apoptosis and klotho, this study still had the limitation that the rat sample size was not large enough. Relevant clinical research is lacking, limited to animal and cell experiments.

In conclusion, our study demonstrated that the chronic androgenized rat model evaluated is useful for studying the long-term effects of androgens on follicular apoptosis. TP might induce both AR expression and klotho expression in the ovary in vivo and in vitro. Exogenous TP induces indirect binding of AR to the klotho promoter to occur abnormal GCs development. Based on the above experimental results, this study proposes that klotho is involved in the apoptosis of GCs and provides a candidate target for the treatment of PCOS.

## Methods

### Materials and reagents

The main reagents are described. Testosterone propionate was produced by AbMole (lot number: T7260); and flutamide was produced by Tokyo Chemical Industry, (F0663, Japan). Dulbecco's modified Eagle's medium (DMEM) and fetal bovine serum (FBS) were obtained from Gibco. Androgen receptor antibody was purchased from Abcam (ab108341), and Klotho antibody was obtained from ABclonal (A12028). The SimpleChIP® Enzymatic Chromatin IP Kit was obtained from CST (#9003).

### Experimental animals

The PCOS model was established using TP [27]. Fifty female Sprague–Dawley rats (48–58 g, 21 days old, investigation number: GLMC-IACUC-2019204.) were obtained from Changsha Tianqin Biotechnology Co., Ltd. (ChangSha, China). Animals were maintained under temperature-controlled (22 °C) conditions on a 12-h light, 12-h dark photoperiod with free access to rat chow and water at animal house of the Clinical Anatomy & Reproductive Medicine Application Institute, Nanhua University.

Twenty-one days old SD rats were randomly divided into three groups (n = 6 in control and vehicle, n = 38 in PCOS). The vehicle group was treated with corn oil (0.1 ml/day for 35 days). In the PCOS group, the PCOS model was established with daily injections of TP (10 mg/kg BW dissolved in corn oil for 35 days, s.c.). From treatment for 21 days, the estrous cycle was monitored by vaginal smears for 2 weeks. After confirming successful establishment of a PCOS model, they were randomly divided into 4 groups (n = 6 in PCOS, n = 8 in other three group). The PCOS-flutamide groups are divided into low-dose group and high-dose group. Flutamide was administered at a dose of 10 mg/kg BW s.c. for the next 15 consecutive days as low dose group and 30 mg/kg BW s.c. is high dose group. All the rats were anesthetized with 0.2 ml/10 g chloral hydrate 24 h after the last treatment. Blood and ovarian samples were collected from all animals. The bilateral ovaries were photographed. Immunohistochemistry, Western blot and TUNEL staining were used for subsequent experiments.

### Cell culture

Ovarian granulosa cells were obtained from BNCC. Cells were cultured in 10% FBS DMEM. Cells were incubated at 37.0 °C and 5% CO_2_ in air. Confluent cells were detached by incubation with 0.05% trypsin for 2 min and inoculated in 96-well plates or 6-well plates containing 10^4^ cells for experiments.

### Western blot analysis

Total protein was extracted, and Western blotting was performed as described previously [28]. Equal quantities of protein lysate (20 µg) were separated on 10% SDS–polyacrylamide and transferred to polyvinylidene difluoride membranes, subsequently, incubated overnight with primary antibody after blocking in non-fat dry milk (5%) and Tris-buffered saline 0.1% tween 20 (TBST) for 1 h. After washing three times with TBST, incubated with secondary antibody for 2 h followed by observing with enhanced chemiluminescence reagents.

### Immunohistochemistry

Briefly, cells were fixed in 4% paraformaldehyde for 30 min at room temperature (RT), subsequently, blocking with endogenous peroxidase solution for 10 min after washing with phosphate-buffered saline (PBS) three times. Cells were incubated overnight with AR antibody (1:100 dilution) in a 4 °C refrigerator, followed by enhancement of the response with an enhancer for 20 min at 37 °C after three washes with PBS. Then, cells were incubated with goat anti-mouse IgG secondary antibody for 20 min at 37 °C after washing with PBS three times, followed by staining for 3 min at RT. Cells were observed under a stereomicroscope.

### CCK-8

GCs cells were seeded into 96-well plate at the density of 1 × 10^4^ cells/100 ml per well with five replicate wells for each sample. Cells were incubated for 24 h after added 5 nmol/L, 25 nmol/L, 50 nmol/L, 75 nmol/L, or 100 nmol/L TP. Cell Counting Kit-8 (CCK-8) (Dojindo, Japan) was used to detect cell viability. 10 μl CCK-8 solution was added to each well under aphotic conditions and further incubated for 2 h. The optical density was measured at 450 nm using a microplate reader (BioTek, USA). The experiment was repeated 3 times under the best conditions.

### Hoechst staining

Cells were seeded in 12-well plates in DMEM with FBS for 24 h. After drug treatment, cells were fixed in 4% paraformaldehyde for 30 min at room temperature and subsequently washed with PBS three times. Then, cells were incubated with Hoechst for 5 min at room temperature after washing with PBS three times. Washing with PBS three times for 30 min. Finally, cells were observed under the microscope after adding the antifade mounting medium.

### Flow cytometry

Cell apoptosis was assessed by an FITC Annexin v Apoptosis Detection Kit (556547, BD Pharmingen ™, USA). Approximately 1.8 × 10^5^ cells/well were reseeded in a 6-well plate. After drug treatment for 24 h or 48 h, we prepared experiments. According to the manufacturer's instructions for apoptosis analysis, cells were separated from 6-well plates with trypsin without EDTA and stained with FITC Annexin v and propidium iodide (PI) positive.

### Chromatin immunoprecipitation (ChIP) assay

In order to cross-link proteins with DNA, each 15 cm Petri dish containing 20 ml of culture medium was added with 1% formaldehyde and incubated for 10 min, and terminated by 2 ml of 10 × glycine. Washing the cells twice with cold PBS and add 2 ml cold PBS + Protease Inhibitor Cocktail (PIC) to obtain the cell pellet. For each IP preparation, resuspend the cells in 1 ml 1 × Buffer A (250 μl 4 × Buffer A # 7006 + 750 μl water) + 0.5 μl 1 M DTT + 5 μl 200 × PIC and place on ice. After 10 min incubation on ice, the cell pellet was obtained and resuspended in 1 ml ice-cold 1 × buffer B (275 μl 4 × Buffer B # 7007 + 825 μl water) + 0.55 μl 1 M DTT + DTT. The centrifugation step was repeated, the supernatant was removed, and the pellet was resuspended in 100 μl of 1 × Buffer B + DTT. Then, 0.5 μl of Micrococcal Nuclease (# 10011) was added and incubated at 37 °C for 20 min. To obtain the precipitate, 10 μl of 0.5 M EDTA was added, and samples were resuspended in 100 μl of 1 × ChIP buffer + PIC and incubated on ice for 10 min. Each immunoprecipitation uses 400 μl 1 × ChIP buffer (40 μl 10 × ChIP buffer + 360 μl water) + 2 μl 200 × PIC. When determining the number of immunoprecipitations, include the positive control sample Histone H3 (D2B12) XP® Rabbit mAb and the negative control sample Normal Rabbit IgG Antibody. The IP samples were incubated at 4 °C for 4 h to overnight. ChIP-grade Protein G Magnetic Beads (# 9006) were resuspended, and 30 μl of Protein G Magnetic Beads was immediately added to each IP reaction and incubated at 4 °C for 2 h with rotation. After washing, the supernatant was obtained, the chromatin was eluted from the antibody/Protein G magnetic beads, and the cross-linking was reversed. After DNA was purified using a spin column, it was quantified by PCR. The primer pairs klotho 115, 5'-tttccctccacagctcagat-3' (forward) and 5'-gtgttacctttcggctctg-3' (reverse), and klotho 177, 5'- ccgagtgggagaaaagtgag-3' (forward) and 5'- ggacgctcaggttcattctc-3' (reverse) were used to amplify the klotho promotor region.

### Statistical analysis

Data were expressed as means ± SD. Statistical differences between groups were applied using Graph Pad Prism software version 7.0 (GraphPad Software, San Diego, CA). All the data were analyzed by T test, data were considered to be statistically significant at *P* value < 0.01 and < 0.05.

## Data Availability

Not applicable.

## Abbreviations

PCOS: Polycystic ovary syndrome
AR: Androgen receptor
TP: Testosterone propionate
GCs: Granulosa cells
CHOP: C/EBP homologous protein
HPG: Hypothalamic-pituitary–gonadal
FBS: Fetal bovine serum
RT: Room temperature
PBS: Phosphate-buffered saline
CCK-8: Cell Counting Kit-8
ChIP: Chromatin immunoprecipitation
IHC: Immunohistochemistry
CNP/NPR2: The natriuretic peptide type C/natriuretic peptide receptor 2
